# Recalcitrant carbon components in glomalin-related soil protein facilitate soil organic carbon preservation in tropical forests

**DOI:** 10.1038/s41598-017-02486-6

**Published:** 2017-05-24

**Authors:** Jing Zhang, Xuli Tang, Siyuan Zhong, Guangcai Yin, Yifei Gao, Xinhua He

**Affiliations:** 1 Key Laboratory of Vegetation Restoration and Management of Degraded Ecosystems, South China Botanical Garden, Chinese Academy of Sciences, Guangzhou, 510650 China; 2 University of Chinese Academy of Sciences, Beijing, 100049 China; 3 School of Environmental Science and Engineering, Guangdong University of Technology, Guangzhou, 510006 China; 4 Centre of Excellence for Soil Biology, College of Resources and Environment, Southwest University, Chongqing, 400715 China; 5 School of Plant Biology, University of Western Australia, Crawley, Australia

## Abstract

Glomalin-related soil protein (GRSP) is known as an important microbial by-product which is crucial for preserving or accumulating soil organic carbon (SOC). However, the underlying mechanisms are not well understood. In this study, we investigated the chemical structures of GRSP and its relationship with SOC using ^13^C nuclear magnetic resonance (NMR) in three tropical forests. The three forests, including a planted forest (PF), a secondary forest (MF) and a primary forest (BF), were selected to represent the natural successional process after disturbance in southern China. Results showed that the average concentrations of GRSP were (3.94 ± 1.09) mg cm^−3^ and accounting for (3.38 ± 1.15)% of the SOC in the top 10 cm soil. NMR analysis indicated rich aromatic C (~30%) and carboxyl C (~40%) in GRSP, and abundant alkyl C (~30%) and O-alkyl C (~50%) in SOC. The recalcitrance indexes (RI), as defined as the ratio of sum of alkyl C and aromatic C over sum of O-alkyl C and carboxyl C, was (98.6 ± 18.9)%, (145.5 ± 10.9)% and (20.7 ± 0.3)% in GRSP higher than that in SOC in the PF, MF and BF, respectively. This study demonstrated that the stubborn structure of GRSP probably regulate the resistance of SOC sequestration in tropical forests, especially in the planted and secondary forests.

## Introduction

Arbuscular mycorrhizal fungi (AMF) form a symbiotic relationship with approximately 80% of the vascular plant species in terrestrial ecosystems^1, 2^. Plants allocate a considerable amount of carbohydrates to arbuscular mycorrhizae (AM)^3^ for exchanging available phosphorus (P) and nitrogen (N)^4, 5^. However, it is controversial whether the carbon (C) allocated to AMF is conducive to SOC accumulation. Several studies have suggested that AMF had a positive effect on SOC accumulation. For example, it was found that the extraradical mycelia of AM, together with vesicles and arbuscules, accounted for 15% of SOC^6^. Fungi- and other microbe-derived C remained in the soil longer than plant-derived C does^7^. In contrast, studies have also found that AMF were negligible and even unfavorable for SOC accumulation^8–11^. Most of these conclusions were based on short-term laboratory experiments under abrupt environmental change scenarios, such as atmospheric CO_2_ enrichment and N addition^8, 10, 11^. For instance, AMF increased organic C decomposition by receiving N that was required by the rapid plant growth under elevated CO_2_ treatment conditions^12^. Notably, soil C dynamics in forest ecosystems had exhibited little fluctuations over time. Therefore, the short-term AMF-mediated increase in C losses may be offset by a long-term C gain in recalcitrant compounds^13^. Moreover, most previous studies have focused on the amounts of C gain or loss via AMF^14–16^, and few has focused on the SOC stabilization processes resulted from AMF.

Most reports on how AMF facilitate SOC accumulation have considered that the external mycelia and its production of glycoprotein, glomalin-related soil protein (GRSP), can promote the formation of soil aggregations^17–19^. The ‘sticky-string-bag’ structure formed by the hyphae, GRSP and the soil particles protects carbonaceous compounds from degrading^17^. Intensive studies have focused on the role of the external mycelia^20, 21^ and soil aggregation^22, 23^ in SOC sequestration. Compared with our understanding about the effects of hyphae and soil particles on SOC sequestration, knowledge on how GRSP promoted SOC accumulation is deficient. Studies have found that GRSP represents a considerable amount (ca. 4–5%) of C in forest soils^24, 25^. The contribution of GRSP to SOC was more than 20 times higher than that of microbial biomass^24^. Although the composition of this glycoprotein was still unclear, Schindler *et al*. found that GRSP had high aromatic C contents^26^, which indicated that it was possible to reveal the mechanism by which GRSP promotes SOC accumulation via the chemical structure of GRSP^26^. Based on these studies, we hypothesized that GRSP promotes SOC sequestration through its high C concentration and its recalcitrant structure. Measurements of the C concentration in GRSP and the chemical structure of GRSP and SOC will provide direct proof of whether and how GRSP facilitates SOC accumulation. The hypothesis is rejected if the dominant component of GRSP is more labile compared to that of SOC.

To detect the chemical structure of GRSP and SOC, ^13^C nuclear magnetic resonance (NMR) was used in three typical unmanaged tropical forest soils. Three forest types were chosen along a successional trajectory, including a planted forest (pine forest, PF), a secondary forest (mixed pine and broadleaf forest, MF), and a primary forest (monsoon evergreen broadleaf forest, BF). Previous work found more severe phosphorus limitation in the BF than in the MF and PF^27^. It was also found higher proportion of C allocated to belowground in the BF compared to the PF when the net primary productivity (NPP) was comparable in these two forests^28^. Therefore, we deduced that the BF probably has more AMF and GRSP than the MF and PF. Furthermore, the BF was found to continuously accumulate SOC^29^, whilst the BF was found to have a higher proportion of non-readily oxidizable organic C in SOC than the PF and the MF^30^. Accordingly, we predicted that the SOC and GRSP have a more recalcitrant C chemical structure in BF than PF. We detected the contribution of GRSP to the SOC and the functional group of the GRSP and SOC in these forests to answer the following questions: (1) Does the contribution of the GRSP to the SOC vary with the successional forest stage? (2) Do the functional groups of the GRSP and/or SOC vary among the forests? (3) Does GRSP have a higher proportion of recalcitrant chemical structures compared to SOC? The answers to these questions will provide insight into the role of GRSP in SOC sequestration.

## Results

Concentrations of both GRSP and SOC were increased from the PF to the MF and the BF. The highest concentrations of GRSP and SOC in 0–10 cm and 10–20 cm soil were found in the BF, followed by the MF and the PF (Table 1). Overall, the mean GRSP contents were (3.94 ± 1.09) and (2.18 ± 0.53) mg cm^−3^ and the mean SOC contents were (23.24 ± 7.82) and (8.29 ± 3.00) mg cm^−3^ in 0–10 and 10–20 cm soil, respectively. The concentrations of both GRSP and SOC were significantly higher in 0–10 cm soil than in 10–20 cm soil in all forests (*P* < 0.05, Table 1).

**Table 1:** Concentrations of GRSP and SOC in the tropical forests of the DBR, southern China.

	Soil depth (cm)	PF	MF	BF
GRSP (mg cm^−3^)	0–10	2.63 ± 0.37 c**	4.22 ± 0.31 b**	4.99 ± 0.57 a**
	10–20	1.56 ± 0.13 b	2.38 ± 0.31 a	2.61 ± 0.33 a
C concentration in GRSP (%)	0–10	19.5 ± 0.88 a**	14.50 ± 1.54 b*	15.47 ± 1.29 b**
	10–20	7.19 ± 0.86 b	9.15 ± 0.27 a	6.83 ± 0.95 b
SOC (mg cm^−3^)	0–10	13.95 ± 1.77 c**	25.01 ± 3.35 b**	30.77 ± 4.39 a**
	10–20	4.76 ± 0.82 c	9.22 ± 2.14 b	10.89 ± 1.16 a

Mean values derived from 7 replications are presented with standard deviations. Different lowercase letters indicate significant differences (*P* < 0.05) among the forests (a, b, c in rows) and * or **indicates significant differences between 0–10 cm and 10–20 cm soils at the *P* = 0.05 or *P* = 0.01 level, respectively. Abbreviations: BF, the primary monsoon evergreen broadleaf forest; MF, the secondary mixed pine and broadleaf forest; PF, the planted pine forest.

### Contribution of GRSP to SOC in tropical forests

**Figure 1:**
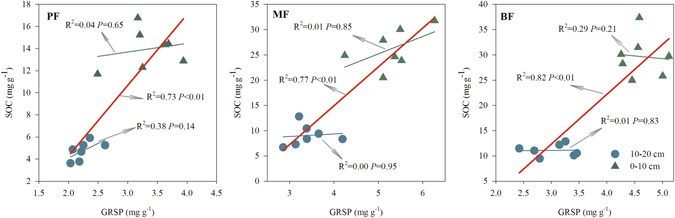
Relationships between glomalin-related soil protein (GRSP) and soil organic carbon (SOC) in the three tropical forests of the Dinghushan Biosphere Reserve, southern China.

To understand the relationship between GRSP and SOC, we plotted the SOC concentration against the GRSP concentration (Fig. 1). The SOC concentration was significantly linearly correlated with the GRSP concentration when combined the samples of 0–10 and 10–20 cm (*P* < 0.01, Fig. 1), which indicated that soil with higher GRSP levels had proportionally more SOC (Fig. 1). However, the relationship between SOC and GRSP was statically insignificant in 0–10 cm or 10–20 cm (*P* > 0.05, Fig. 1), which could probably be explained by the different order of magnitude of SOC and GRSP (Fig. 1).

We detected the C concentrations in GRSP for all the 42 samples collected from these tropical forests. The C concentration in GRSP was (19.5 ± 0.88)% in the PF in 0–10 cm soil, which was significantly higher than those in the MF and BF at the same depth (*P* < 0.05, Table 1). The C concentration in the GRSP in 10–20 cm soil was significantly lower than those in 0–10 cm soil (*P* < 0.05, Table 1). The mean C concentrations in the GRSP in 10–20 cm soil were (7.19 ± 0.86)%, (9.15 ± 0.27)% and (6.83 ± 0.95)%, which accounted for 63%, 37% and 56% of the C concentrations in the GRSP in 0–10 cm soil in the PF, MF, and BF, respectively.

**Figure 2:**
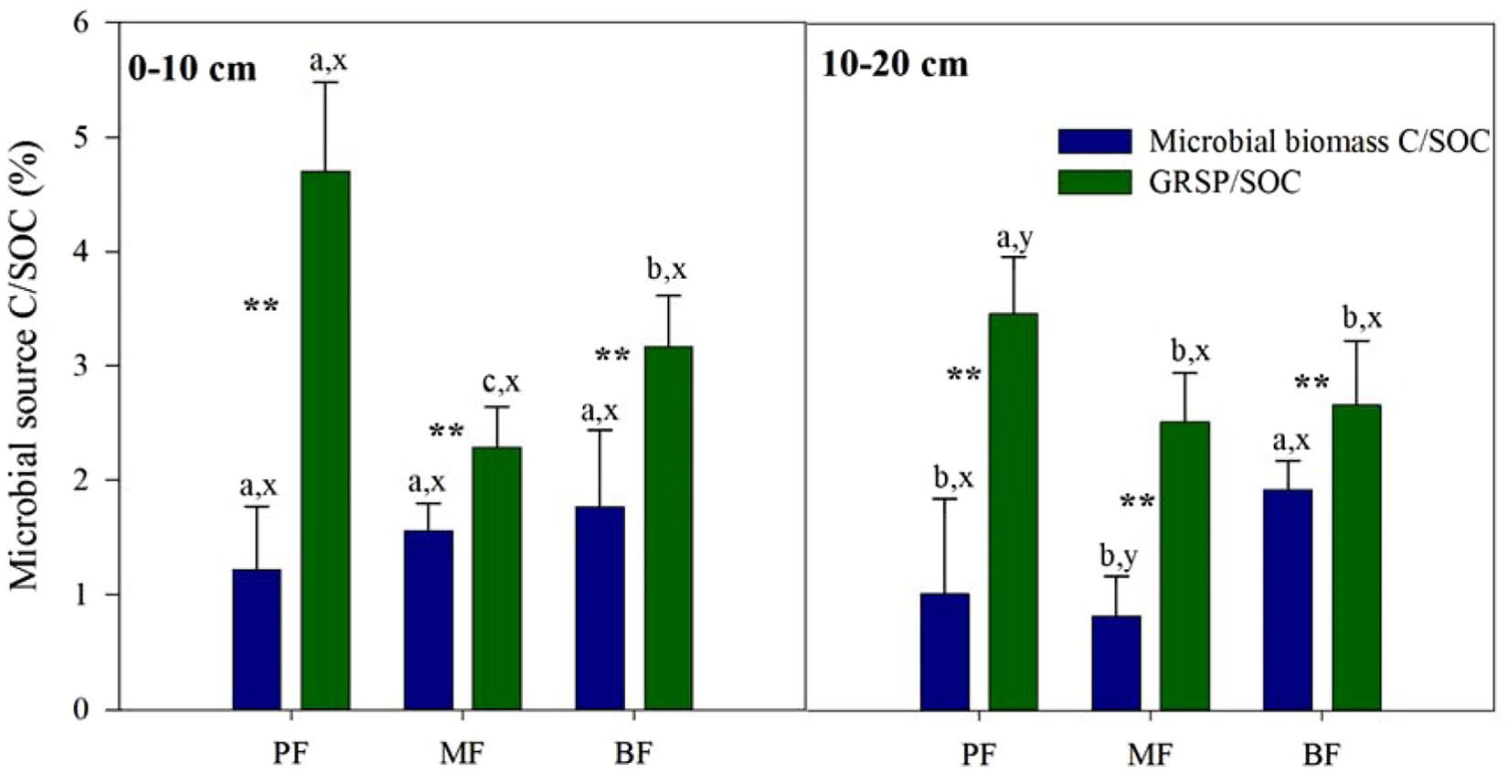
Contributions of microbial biomass C and GRSP to SOC in 0–10 cm soil and 10–20 cm soil in the tropical forests of DBR, southern China. Mean values with standard deviations of 7 replications are presented. Different letters indicate significant differences (*P* < 0.05) between the forests (a, b, c) and between the soil depths (x, y), and * or ** indicates significant differences between the contribution of the microbial biomass-C/SOC and GRSP/SOC at the *P* = 0.05 or *P* = 0.01 level, respectively.

The contribution of GRSP to the SOC, as indicated by the GRSP to SOC ratio (GRSP/SOC), was (4.70 ± 0.77)% in the PF, which was 2.1 and 1.6 times greater than those in the MF and the BF in 0–10 cm soil (Fig. 2). The GRSP/SOC ratio in 10–20 cm soil in the PF was also remarkably higher than those in the MF and the BF (*P* < 0.05, Fig. 2). In all forests, the contribution of GRSP to SOC was significantly higher than that of the soil microbial biomass C (*P* < 0.01, Fig. 2). The soil microbial biomass C accounted for (1.22 ± 0.55)% (PF) to (1.76 ± 0.45)% (BF) of the SOC (Fig. 2). The GRSP/SOC was 1.61–6.73 times greater than the soil microbial biomass-C/SOC in the tropical forests.

### Chemical structure of GRSP and SOC

**Figure 3:**
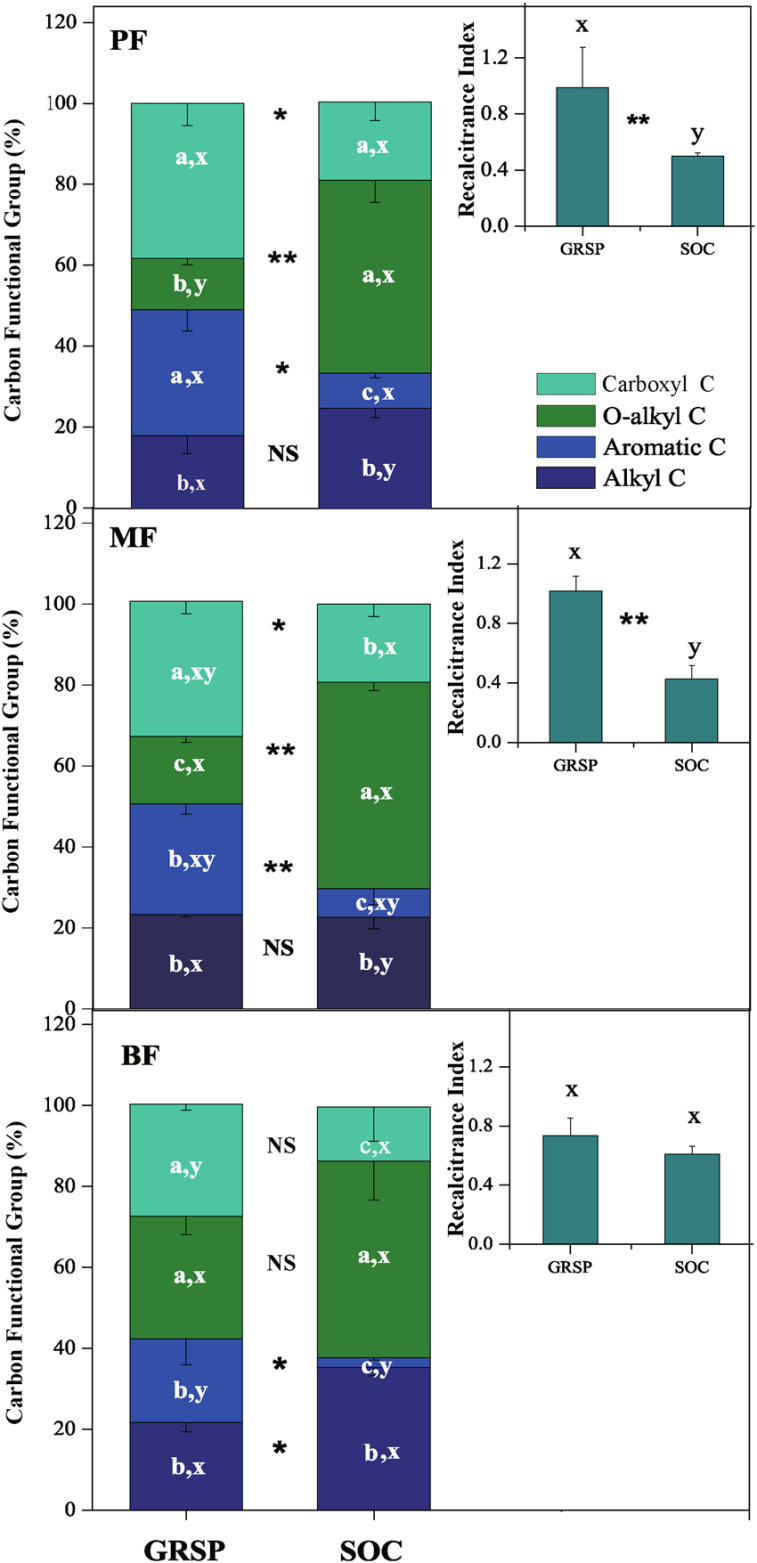
Distributions of functional groups of GRSP and SOC in the three tropical forests in DBR, southern China. Data are presented as means with deviations of 7 replications. The inserted figures present the recalcitrance indexes, indicated by (alkyl C + aromatic C)/(O-alkyl C + carboxyl C), of GRSP and SOC. Different letters indicate significant differences (*P* < 0.05) between the C functional groups (a, b, c) and between the forests (x, y, z), and * or ** indicates significant differences in the C functional groups or recalcitrance index between GRSP and SOC at *P* = 0.05 or *P* = 0.01 level, respectively.

The chemical structures of GRSP and SOC detected using solid-state ^13^C cross polarization magic angle spinning (CPMAS) NMR spectra are presented in Fig. 3. GRSP and SOC had different C functional groups in the tropical forest soils. Overall, the SOC had high proportion of O-alkyl C (~50% of total) and low aromatic-C proportion (<10% of total), while the GRSP had high carboxyl C proportion (~40% of total) and relatively high aromatic C proportion (~30% of total) and alkyl C proportion (~20% of total).

The recalcitrance index, indicated by (alkyl C + aromatic C)/(O-alkyl C + carboxyl C)^31^, was higher in GRSP than in SOC (*P* < 0.05, Fig. 3). In the PF and the MF, GRSP had higher proportion of aromatic C and comparable proportion of alkyl C compared to SOC. In the BF, GRSP had 18.33% higher proportion of aromatic C and 13.66% significant lower alkyl C proportion compared with SOC (*P* < 0.05, Fig. 3). Accordingly, GRSP had higher recalcitrance index than SOC, although this difference was not statistically significant in the BF (Fig. 3).

**Figure 4:**
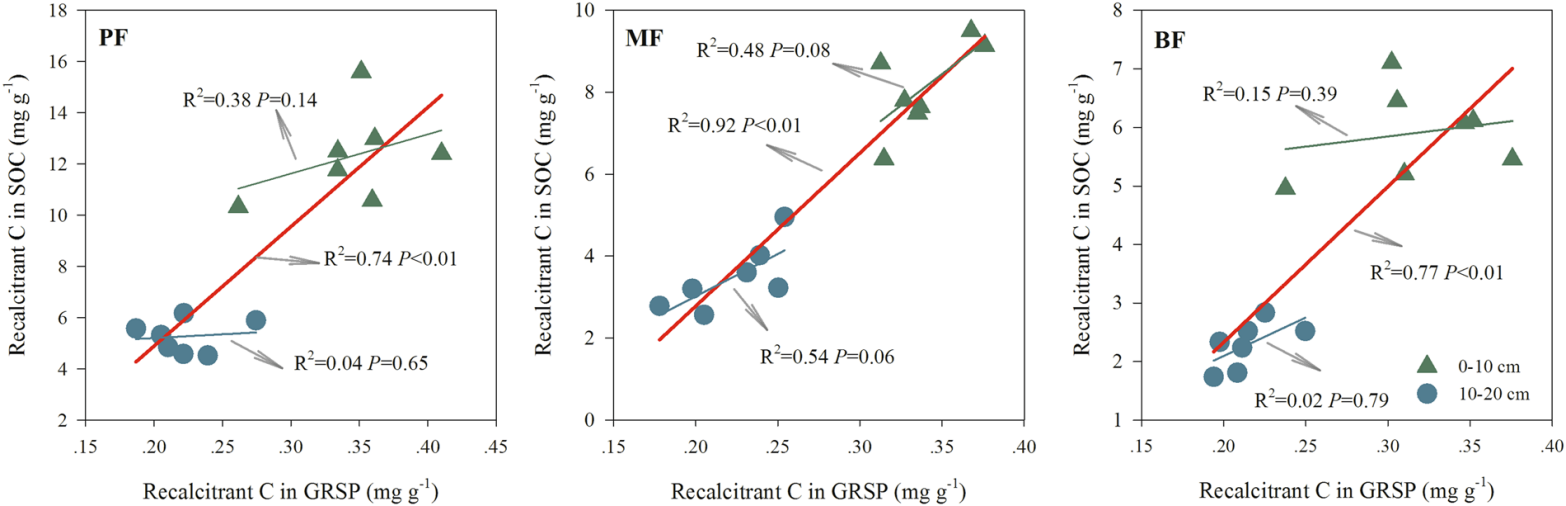
The relationship between the recalcitrant C concentrations (mg g^−1^) in the SOC and GRSP in the tropical forests of DBR, southern China.

Recalcitrant C (alkyl C + aromatic C) in GRSP presented a significantly positive correlation with the recalcitrant C in SOC when taken data from 0–10 and 10–20 cm soils together although the relationship was insignificantly in each soil depth (Fig. 4). Variation in the recalcitrant C in GRSP explained 77% of the recalcitrant C variations in SOC in the BF, 92% in the MF, and 74% in the PF (*P* < 0.01, Fig. 4).

## Discussion

The concentrations of GRSP (2.03–6.26 mg g^−1^, 0–10 cm soil) in the tropical forests were lower than those in a tropical forest in Costa Rica (1–27 mg g^−1^, 0–10 cm soil)^25^ and in a secondary rainforest in the French West Indies (2–36 mg g^−1^, 0–10 cm soil)^32^, but were higher than those in citrus plantation in the northern subtropical China (0.7–1.33)^33^. Multiple factors, including climatic factors (i.e., temperature and precipitation), fungi related factors (i.e., fungal type, diversity, biomass, etc.), and host plant traits (i.e., species, diversity, growth rate, etc.), and their interactions determine the different GRSP concentrations observed across the various study sites^17, 24, 34^. We suggested that the lower concentration of GRSP in our study site than in Costa Rica and in the French West Indies is explained by the lower SOC content, the relatively lower annual mean precipitation and temperature, and species diversity etc. in our study sites compared with those tropical forests (Table S1). While the higher GRSP level in our study site compared with that in the citrus plantation in northern subtropical China is probably be resulted from the higher SOC content, annual precipitation, temperature and species diversity in our study sites than in the citrus plantation in northern subtropical China (Table S1). In our study site, the GRSP concentrations were increased with the successional processes, as indicated by the highest GRSP concentration in the BF and the lowest concentration in the PF (Table 1). The GRSP concentration in the three successional forests showed a tendency of a higher mycorrhizal colonization ratio, leading to richer mycorrhizal plant species^35^.

The C concentration in the GRSP in 0–10 cm soil (12.95–20.2%) in the three tropical forests was comparable to those in Hawaiian forest soils at the same soil depth (9.9–22.0%)^24^ but were lower than those in tropical forests in Costa Rica (~37%)^25^. Soil properties, such as the bedrock type and fertility, explained the differences in the C concentrations in the GRSP across sites^24, 36^. The large variance of the C concentration in GRSP found in different studies also demonstrated that GRSP was not a specific protein but a mixture instead, such as humus and fulvic acid^17^.

The positive relation between GRSP and SOC (Fig. 1) found in this study was in accordance with the results of trifoliate orange pot^37^ and maize field experiments^4^. The insignificant correlation between GRSP and SOC in each soil depth intervals (i.e., 0–10 cm and 10–20 cm soils) was explained by the SOC concentration and the GRSP concentration varied in different orders of magnitude (4–40 mg g^−1^ *vs* 2.0–5.0 mg g^−1^). Similar tendency was found in tropical soil chronosequence^24^, where the GRSP concentration was insignificance correlated with the SOC concentration for either O horizon or A horizon (Fig. S1a). However, significant positive relationship was found between the GRSP concentration and the SOC concentration when taken O horizon and A horizon into consideration together (Fig. S1a). A further analysis with data derived from cropland^4, 38^, cooper pollution area^39^, and forests^24^ indicated substantial variation with the positive correlation between the GRSP concentration and the SOC concentration (Fig. S1b). The variation could be the spatial heterogeneity across different studying regions, as well as the different order of magnitude of the SOC and the GRSP concentration.

The contribution of GRSP to SOC (GRSP/SOC) found in tropical forests (Fig. 2) was comparable to those found in grassland (3.77–7.84%)^40, 41^, farmland (2.79–6.97%)^42, 43^ and forests (4–5%)^24, 25^. GRSP accounted for 8–30% of the SOC in the Mu Us sandland, where the SOC is relatively low (~0.2–1%)^44^. Higher GRSP/SOC ratios were consistently found in unfertilized soils^45^, showing that ecosystems with infertile soil are probably more dependent on AMF to obtain N or P than in an ecosystem with fertile soil^1^. In our study sites, the higher GRSP/SOC values in the PF were consistent with the lower soil C, N, and P levels in this forest compared with those in the MF and the BF (Tables 1 and 2). In contrast, the relatively low GRSP/SOC in the MF and BF could be explained by the high SOC level in these two forests (Table 1). Furthermore, the higher GRSP concentration and low GRSP/SOC in the MF and the BF revealed that these forests have diverse soil C sources compared with the PF. However, in temperate forests, Jorge-Arager *et al*. found that the GRSP/SOC level (~2%) was irrelevant to the site conditions due to the narrow range of GRSP and SOC in all 10 sampling sites^46^. Other studies in tropical forests have also found obvious variations in the GRSP/SOC across sites^32^, likely indicating that the GRSP/SOC is also altered by differences in the plant and AMF compositions^21, 47^.

**Table 2:** Characteristics of tropical forests and soils (0–10 cm soil) in the DBR, southern China.

Forest types	PF	MF	BF	References
Biomass (Mg ha^−1^)	127.01	271.30	273.99	Unpublished data from Dinghushan Forest Ecosystem Research Station, 2010.
Fine root biomass (Mg ha^−1^)	1.9 ± 1.1 b	2.8 ± 1.1 ab	4.9 ± 3.0 a	
Diversity (numbers of tree species ha^−1^)	25	54	92	
Density (numbers of individual trees ha^−1^)	920	4740	5581	
AMF plant ratio (%)	87.5	76.9	90.9	Measured in this study
Soil water content (%)	16.4 ± 1.2 c	25.9 ± 2.0 b	28.1 ± 1.3 a	
Soil pH	3.81 ± 0.06 b	3.91 ± 0.07 a	3.66 ± 0.05 c	
Total N (mg g^−1^)	1.31 ± 0.14 c	2.11 ± 0.19 b	2.57 ± 0.24 a	
Total P (mg g^−1^)	0.14 ± 0.06 b	0.14 ± 0.01 b	0.24 ± 0.01 a	
Ammonium N (mg kg^−1^)	4.08 ± 0.45 b	7.45 ± 0.84 a	5.37 ± 0.46 b	
Nitrate N (mg kg^−1^)	19.41 ± 2.67 b	25.68 ± 1.59 a	27.06 ± 5.52 a	
Available P (mg kg^−1^)	1.73 ± 0.36 ab	1.27 ± 0.27 b	1.81 ± 0.21 a	

Mean values with standard deviations derived from 7 replications are presented in fine root biomass and soil parameters. Different lowercase letters indicate significant differences among the forests (*P* < 0.05).

As a microbe-derived C pool^24^, GRSP made a substantial contribution to the SOC, which was 1.6 to 6.7 times higher than the contribution of microbial biomass C (Fig. 2). The fact that the GRSP/SOC ratio was much higher than the microbial biomass-C/SOC ratio indicated that the contribution of GRSP to SOC exceeds the contribution of the standing AMF hyphae biomass C, which is part of the microbial biomass C. Studies have shown that the average lifespan of GRSP (6–42 years)^24^ is remarkably prolonged than the mean residence time of hyphae (5–6 days)^15, 48^. Relatively high GRSP concentrations and high GRSP/SOC ratio highlighted the importance of AMF production in soil C accumulation. Moreover, our results indicated that AMF is a larger C sink in tropical forest soils than previously assumed based on live extraradical or intraradical hyphae^49^.

The solid-state ^13^C CPMAS NMR spectra of GRSP indicated a comparable proportion of aromatic C and O-alkyl C in tropical forests (Fig. 3). The proportion of aromatic and O-alkyl C concentrations of GRSP ranged from 17% to 34% and from 11% to 33%, respectively, for the three forests. Compared to the results of a study conducted in eastern South Dakota, USA (42–49% of aromatic C, 24–30% of carboxyl C, 4–14% of alkyl C and 4–16% of O-alkyl C)^26^, the GRSP observed in our forests had a lower aromatic C but higher carboxyl, alkyl and O-alkyl C proportions. Schindler *et al*. suggested that GRSP possessed a similar C-type distribution to that of humic acid, although the chemical structure of GRSP is still unclear^26^. Therefore, further study is expected to detect the chemical structure of GRSP, and GRSP may represent a material with rich aromatic and carboxyl C.

A higher recalcitrance index of SOC in the BF than in the MF and the PF indicated that SOC was more stable in the BF than in the PF and the MF (Fig. 3), which was in accordance with the increase tendency of the non-readily organic C from the PF to the BF^30^. A relatively high recalcitrance index of SOC in the BF is the result of the high proportion of alkyl C (Fig. 3). Alkyl C in the soil originates from lyophobic materials, such as methyl, waxes, cutins, lipids, and other components, which are extremely difficult to decompose^50, 51^. A greater litter amount and decomposition rate may explain the recalcitrant C accumulation in the BF^52^. Lower aromatic C in the BF than in the PF is partly determined by less coniferous trees in the BF. Pine needles contain phenols and tannin components, which are the aromatic C source in soil^28, 53^. Previous studies have found that the alkyl C and aromatic C concentrations of SOC vary among sites. For example, higher alkyl (~60%) and lower aromatic C (~19%) levels of the SOC were observed in an untouched forest soil in the northern part of the Subpolar Urals^54^ and a significantly lower alkyl C (~10%) and higher aromatic C (~30%) of SOC were measured in the soil of Spanish oak forests^55^. Different alkyl C and aromatic C proportions among the study sites are probably explained by differences in plant functional types, microbial community structure, climate, and soil parent material. Any difference in these factors could alter the input and the turnover rate of SOC, resulting in different levels of SOC composition among study sites.

The significantly higher recalcitrance index of GRSP than SOC (Fig. 3) and the linear relationship between the recalcitrant C in GRSP and SOC (Fig. 4) suggested that GRSP probably promoted SOC sequestration in tropical forests. Previous studies have indicated that GRSP is an important part of slow or passive soil C because of its long residence time (~11–92 years) in soils^38^. The fact that the recalcitrance index of GRSP is much higher than that of SOC (Fig. 3) indicates that GRSP is vital for SOC sequestration. The highest GRSP/SOC ratio together with the significantly higher recalcitrance index of GRSP than SOC (Fig. 3) in the PF suggested that GRSP was more important for SOC preservation in the PF than in the MF and the BF. Different recalcitrance characteristics of GRSP and SOC in these three forests indicated that the superiority of the AMF in the PF was gradually weakened in the MF and BF. Moreover, combining the sticky nature of GRSP to the soil aggregation formation^17–19^, we can conclude that GRSP plays an important role in SOC accumulation directly and indirectly. GRSP facilitates SOC accumulation directly due to the amount of C it retains and indirectly by the recalcitrant structure of the prolonged organic C reserved in soils.

## Materials and Methods

### Study area

The study site is located in the Dinghushan Biosphere Reserve (DBR, 23°10′N, 112°32′E, 200–300 m above sea level, Guangdong, southern China), which was established in the 1950s with an area of 1,200 ha to protect the undisturbed natural monsoon evergreen broadleaf forest (BF)^29^. The DBR has a typical tropical monsoon climate, with a mean annual precipitation of 1,927 mm (80% between April and September). The annual mean temperature is 22.3 °C, with a monthly mean temperature of 28 °C in July and 12.6 °C in January. The bedrock is sandstone and shale of Devonian age. The soil is classified in the Ultisols group and Udult subgroup (USDA Soil Taxonomic System), with a pH of 3.6–3.9 in the top 10 cm depth^56^. The basic soil physicochemical properties, biomass and the mycorrhizal type under the three forest conditions are presented in Table 2, and further information on the three forests has been described by Tang *et al*.^28^ and Huang *et al*.^27^.

### Field sampling

In December 2012, seven 20 × 20 m subplots were randomly arranged in each 10,000 m^2^ permanent plot for soil sampling. With a 2.5 cm diameter auger, 8 soil cores at 0–10 and 10–20 cm soil were randomly collected as one composite soil sample in each plot. Soil samples were thoroughly mixed and put into plastic bags, stored in iceboxes, then transported to the laboratory for analysis. Each composite sample (~700–800 g) was divided into two subsamples. One fresh subsample was used for microbial C analysis, and the other was air dried for GRSP and soil physicochemical property analysis.

### Laboratory analysis

#### GRSP extraction and purification

The determination of the GRSP was conducted according to an improved protocol based on the Bradford protein assay^41, 57^. To assess the C concentration and functional groups of the GRSP, the crude extracted GRSP was purified according to the following procedures^58^: The GRSP extraction was subjected to precipitation by 1.0 mol L^−1^ hydrochloric acid, then centrifuged at 10,440 rpm for 10 min after incubation in ice for 1.0 h^58^, and then precipitated again by 0.1 mol L^−1^ sodium hydroxide (NaOH) to ensure that all the precipitation was reconstituted. The precipitated samples were transferred into dialysis tubing (≤12,000 Daltons) and placed in dH_2_O for dialyzing at 6–8 h intervals 4 times under conditions of constant stirring. The purified dialysate was centrifuged at 10,440 rpm for 10 min to remove any extraneous particles, and the collected supernatant was frozen at −20 °C and then dried in a vacuum freeze drier.

#### Solid-state ^13^C NMR spectra

Before the NMR spectra detection, 10 g of each air-dried soil sample was pre-treated with hydrofluoric acid as described in detail in Mathers *et al*.^50^. The ^13^C cross polarization magic angle spinning (CPMAS) NMR values of purified GRSP and pre-treated soil were obtained at a frequency of 22.62 MHz on an AVANCE III spectrometer (Bruker Ascend^TM^ 300 WB, Bruker, Karlsruhe, Germany). Samples were placed into a 4 mm CPMAS rotor and spun at 5000 Hz at the magic angle. With a frequency rate of 7547 Hz and a recycle delay of 2.5 s, the single contact time of 2 ms was applied. Approximately 5000 scans for soil or 20,000 scans for GRSP transients were collected from each sample. The ^13^C NMR spectra were integrated based on the chemical shift value: 0–50 ppm (alkyl C), 50–110 ppm (O-alkyl C), 110–160 ppm (aromatic C) and 160–220 ppm (carboxyl C)^50, 59^.

The stability of SOC was determined with the recalcitrance index, which is the ratio of (alkyl + aromatic C)/(O-alkyl + carboxyl C)^31^. Alkyl and aromatic compounds include long-chain aliphatics and tannin, among other compounds, which are hydrophobic and resist decay, whereas O-alkyl and carboxyl C represent compounds such as organic acids, which are hydrophilic and labile^31^. To evaluate the contribution of recalcitrant C, the concentration of SOC or GRSP multiplying the recalcitrance index of SOC or GRSP was calculated to explore the relationship between the recalcitrant C of GRSP and soil, respectively.

#### Soil physicochemical properties and microbial biomass

The soil pH was measured with a ratio of 1:2.5 (w/v) of soil to deionized water using a pH meter. The SOC was detected by titration with 0.2 mol L^−1^ FeSO_4_ solution after dichromate oxidation^60^. The available P was extracted using an acid-ammonium fluoride solution (0.03 mol L^−1^ NH_4_F and 0.025 mol L^−1^ HCl)^60^. Ammoniacal N was measured using the indophenol blue method followed by colorimetry, and nitric N was determined after cadmium reduction to nitrite, followed by the sulfanilamide-NAD reaction^60^. Microbial biomass C was determined using the chloroform fumigation-extraction method^61^.

To better understand the contribution of the GRSP to the SOC, part of the freeze-dried GRSP samples was collected through a 0.053 mm sieve and analyzed for the C concentration on the preprocessor of a stable isotope ratio mass spectrometer (IsoPrime 100, IsoPrime, UK). The contribution of GRSP to SOC (expressed as GRSP/SOC) was calculated with Equation (1):

1 $$GRSP/SOC=\frac{GRSP-C\times [GRSP]}{[SOC]}$$

where GRSP/SOC and GRSP-C denote the contribution of GRSP to SOC, the C concentration in GRSP, respectively. [GRSP] and [SOC] are the concentrations of GRSP and SOC, respectively.

#### Data analysis

Data were checked for normal distributions and homogeneity of variances before significance analyses. A series of one-way ANOVAs followed by the least-significant difference (LSD) test was used to test for significant differences in the GRSP, SOC and C concentration of GRSP across forests at the *P* < 0.05 level. Significant differences between the GRSP/SOC and microbial biomass C/SOC were evaluated using a paired *t* test for each forest. The paired *t* test was also performed to examine differences in the functional group between the GRSP and SOC in each forest type. The relationships between GRSP and SOC and between the recalcitrant C in GRSP and in SOC were analyzed using linear regression. All statistical analyses were performed using SPSS 20.0 (Statistical Package for the Social Sciences, Chicago, IL, USA).

## Electronic supplementary material

Supplementary materials
