# Supplementary material for: Recalcitrant carbon components in glomalin-related soil protein facilitate soil organic carbon preservation in tropical forests
Source: Sci Rep. 2017 May 24;7:2391. doi: 10.1038/s41598-017-02486-6 (PMC5443815; doi:10.1038/s41598-017-02486-6)
Supplement: Supplementary file 1 — Supplementary materials [file 41598_2017_2486_MOESM1_ESM.doc]

**Recalcitrant carbon components in glomalin-related soil protein facilitate soil organic carbon preservation in tropical forests**

**Jing Zhang^1,2^, Xuli Tang^1*^, Siyuan Zhong^1,2^, Guangcai Yin^3^, Yifei Gao^1,2^ & Xinhua He^4, 5^**

^1^ Key Laboratory of Vegetation Restoration and Management of Degraded Ecosystems, South China Botanical Garden, Chinese Academy of Sciences, Guangzhou 510650, China

^2^ University of Chinese Academy of Sciences, Beijing 100049, China

^3^ School of Environmental Science and Engineering, Guangdong University of Technology, Guangzhou 510006, China

^4^ Centre of Excellence for Soil Biology, College of Resources and Environment, Southwest University, Chongqing 400715, China

^5^ School of Plant Biology, University of Western Australia, Crawley, Australia

**Supporting Information**

**Supporting Figure S1**

**Figure S1 Relationships between the GRSP concentration and the SOC concentration in different soil layers of chronosequence tropical forests (panel a) and in different study sites (panel b).** (a)The relationship between the GRSP concentration and the SOC concentration in O horizon and A horizon in chronosequence tropical soils, data were derived from Table 3 in Rillig et al^1^. (b) The relationship between GRSP and SOC in different study sites including cropland (Preger et al.^2^, pink cycles; Koide et al.^3^, red cycles), cooper pollution area (Cornejo et al.^4^, blue cycles), and tropical forests (Rillig et al.^1^, green cycles). All data were Z transformed before analysis.

**
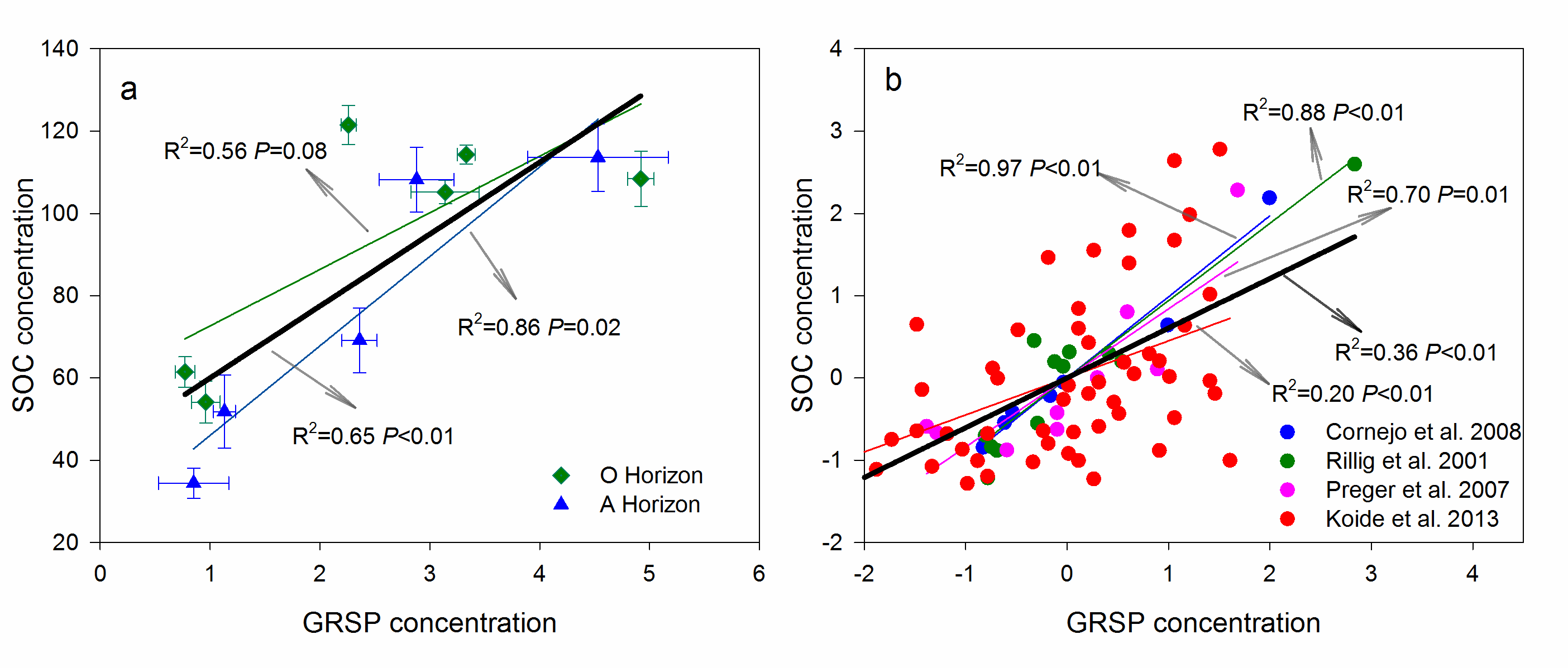
**

**Supporting Table S1**

**Table S1. The comparison of site conditions between different studies.**

| Study Site | GRSP content (mg g^-1^) | Forest type | Annual precipitation (mm) | Annual mean temperature  (°C) | Numbers of tree species | Mycorrhizal colonization rate (%) | Dominant mycorrhizae type | Soil organic carbon content (mg cm^-3^) | pH |
| --- | --- | --- | --- | --- | --- | --- | --- | --- | --- |
| NE Costa Rica^5^ | 1-27 | Tropical wet forest | 4000 | 26 | 320 | - | AM | 29.8-50.3 | 4.00-4.52 |
| French West Indies^6^ | 24.5±8.7 | secondary rainforests | 6000 | 26-28 | - | - | AM | 40-112 | 4.5-6.7 |
| Northern subtropical China ^7^ | 0.7-1.33 | *Citrus unshiu* trees | 1100-1300 | 16.2-16.6 | 1 | 17-51 | AM | 9.4 | 6.2 |
| Southern China (this study) | 2.03-6.26 | Pine forest, mixed forest and monsoon evergreen broadleaved forest | 1927 | 22.3 | 25-92 | 20-40 | AM^8^ | 12.18-36.16 | 3.61-3.97 |

**References**

1 Rillig, M. C., Wright, S. F., Nichols, K. A., Schmidt, W. F. & Torn, M. S. Large contribution of arbuscular mycorrhizal fungi to soil carbon pools in tropical forest soils. *Plant Soil* **233**, 167-177 (2001).

2 Preger, A. C. *et al.* Losses of glomalin-related soil protein under prolonged arable cropping: A chronosequence study in sandy soils of the South African Highveld. *Soil Biol. Biochem.* **39**, 445-453, doi:10.1016/j.soilbio.2006.08.014 (2007).

3 Koide, R. & Peoples, M. Behavior of Bradford-reactive substances is consistent with predictions for glomalin. *Applied Soil Ecology* **63**, 8-14, doi:10.1016/j.apsoil.2012.09.015 (2013).

4 Cornejo, P., Meiera, S., Borie, G., Rillig, M. C. & Borie, F. Glomalin-related soil protein in a Mediterranean ecosystem affected by a copper smelter and its contribution to Cu and Zn sequestration. *Sci. Total Environ.* **406**, 154-160, doi:10.1016/j.scitotenv.2008.07.045 (2008).

5 Lovelock, C., Wright, S., Clark, D. & Ruess, R. Soil stocks of glomalin produced by arbuscular mycorrhizal fungi across a tropical rain forest landscape. *J. Ecol.* **92**, 278-287, doi:10.1111/j.0022-0477.2004.00855.x (2004).

6 Woignier, T., Etcheverria, P., Borie, F., Quiquampoix, H. & Staunton, S. Role of allophanes in the accumulation of glomalin-related soil protein in tropical soils (Martinique, French West Indies). *Eur. J. Soil Sci.* **65**, 531-538, doi:10.1111/ejss.12151 (2014).

7 Wu, Q.-S., Wang, S., Cao, M.-Q., Zou, Y.-N. & Yao, Y.-X. Tempo-spatial distribution and related functionings of root glomalin and glomalin-related soil protein in a citrus rhizosphere. *Journal of Animal and Plant Sciences* **24**, 245-251 (2014).

8 Zheng, K., Tang, X., Zhang, J. & Han, T. Mycorrhizae respond to plant diversity in monsoon evergreen broadleaved forest succession choronsequence. *Ecology and Environmental Sciences* **22**, 729-738 (2013).
